# Nonlinear XFEM Modeling of Mode II Delamination in PPS/Glass Unidirectional Composites with Uncertain Fracture Properties

**DOI:** 10.3390/ma13163548

**Published:** 2020-08-12

**Authors:** Damoon Motamedi, Mahdi Takaffoli, Abbas S. Milani

**Affiliations:** 1 School of Engineering, University of British Columbia, Kelowna, BC V1V 1V7, Canada; damoonmotamedi@gmail.com; 2 Materials and Manufacturing Research Institute, University of British Columbia, Kelowna, BC V1V 1V7, Canada; mahdi.takaffoli@ubc.ca

**Keywords:** composites, stochastic fracture behavior, end notched flexure testing, nonlinear extended finite element model

## Abstract

Initiation and propagation of cracks in composite materials can severely affect their global mechanical properties. Due to the lower strength of the interlaminar bonding compared to fibers and the matrix, delamination between plies is known to be one of the most common failure modes in these materials. It is therefore deemed necessary to gain more insight into this type of failure to guide the design of composite structures towards ensuring their robustness and reliability during service. In this work, delamination of interlaminar bonding in composite end-notched flexure (ENF) samples was modeled using a newly developed stochastic 3D extended finite element method (XFEM). The proposed numerical scheme, which also incorporates the cohesive zone model, was used to characterize the mode II delamination results obtained from ENF testing on polyphenylene sulfide (PPS)/glass unidirectional (UD) composites. The nonrepeatable material responses, often seen during fracture testing of UD composites, were well captured with the current numerical model, demonstrating its capacity to predict the stochastic fracture properties of composites under mode II loading conditions.

## 1. Introduction

Nowadays, fiber reinforced polymer (FRP) composites are widely used in various engineering applications, including aeronautical, marine, and automotive industries. These materials have high strength-to-weight ratios as well as good corrosion resistance and can be engineered based on the required strength or performance objectives of a given application. Although FRP composite structures have proven to provide numerous advantages, initiation and propagation of cracks in these materials can drastically affect their mechanical properties. The most common failure modes in these composites are classified as fiber breakage, fiber pull-out, matrix cracking, and interlaminar delamination. Among these, interlaminar delamination is perhaps the most common type and may occur because of weak bonding between composite layers. This failure mechanism can significantly reduce the structural stiffness of FRP structures and weaken their tensile or shear capacity under service loads [1].

To improve the mechanical performance of FRP composites in the presence of process-induced or loading-induced cracks, extensive studies on their fracture properties have been performed, both experimentally and numerically [2,3,4,5]. Gaggar and Broutman [2] utilized both single and double edge-notched tensile tests as well as a notched bend test to extract the critical stress intensity factors of these cracks in composite materials. Mower and Li [3] summarized the experimental results from previous investigations and concluded that linear elastic fracture mechanics (LEFM) is not a valid approach for long-fiber composites and that a nonlinear material constitutive model is instead required to accurately characterize their fracture response. In an attempt to measure mode II fracture toughness, Russel [4] proposed end-notched flexure (ENF) testing. Murri and O’Brien [5] employed ENF samples to study the mode II fracture toughness of FRP composites while investigating the error associated with neglecting nonlinear terms in the calculation of strain energy release rate. ENF testing is currently one of the most recognized experimental methods for studying the mode II fracture of FRP composite materials.

Numerical models of crack initiation and propagation during ENF testing of composites have also been developed in the past (e.g., Harper and Hallett [6] and Fan et al. [7]) to facilitate the design of composite structures prior to prototyping and testing stages. However, these investigations do not typically account for the heterogeneous and stochastic properties of composites, which are commonly observed during material and product testing. There can be a variety of sources for such nonuniformity of material properties in composites. Examples include random distributions of fibers within samples, fiber penetration between layers, existence of voids within the matrix, human error in manufacturing process, and uneven heating or cooling of samples during molding. Adopting deterministic approaches and ignoring the spatial variability that exists in composites can introduce errors into large-scale simulations. Consequently, stochastic modeling of effective material properties appears to be essential for more precise assessment of the mechanical behavior of composites, especially during the prediction of critical failure loads and crack formation patterns [8,9].

Among recent stochastic modeling works in the field, Ashcroft et al. [10] introduced microstructure randomness in the fracture properties of carbon fiber reinforced polymer composite materials for finite element simulation of double cantilever beam (DCB) tests using interface cohesive elements. Nonuniformity and random distribution of material fracture properties were considered by means of uniform and Weibull distributions. Jumel [11] employed a finite difference numerical method to study crack initiation and propagation in DCB specimens with randomly fluctuating interface properties along the crack path. The effect of this variability at the microscopic level on the parameters measured at the macroscopic level was investigated. The results emphasized the need for further development of computational approaches that account for randomness and variability in material properties.

The present study was aimed at developing and examining an enhanced numerical approach for simulating fracture in unidirectional (UD) composite structures by considering both material and geometric nonlinearities along with stochastic fracture properties. An Abaqus user element subroutine was developed and linked to MATLAB to model under ENF testing (mode II fracture). Here, we adopted the extended finite element method (XFEM) and enhanced it with contact and cohesive zone modeling capabilities along with stochastic fracture properties to improve the simulation of crack propagation in the composite laminates. The cohesive zone model constituted a bilinear traction–separation law at the crack front, which enabled modelling of the fiber bridging mechanism in the process zone ahead of the crack tip during loading. Stochastic distributions of the fracture properties were captured within the bilinear traction–separation law. The numerical results were compared with a set of tests performed on polyphenylene sulfide (PPS)/glass UD composites.

## 2. Experimental

PPS/glass UD composite samples comprising 14 plies and with nominal dimensions of 250 mm length, 25 mm width, and 3 mm thickness were prepared for ENF testing. To introduce an edge crack with a nominal size of 43 mm in each sample, a polyimide Teflon sheet was placed between the middle layers of the stacked laminates before placing them in the oven for curing.

**Figure 1:**
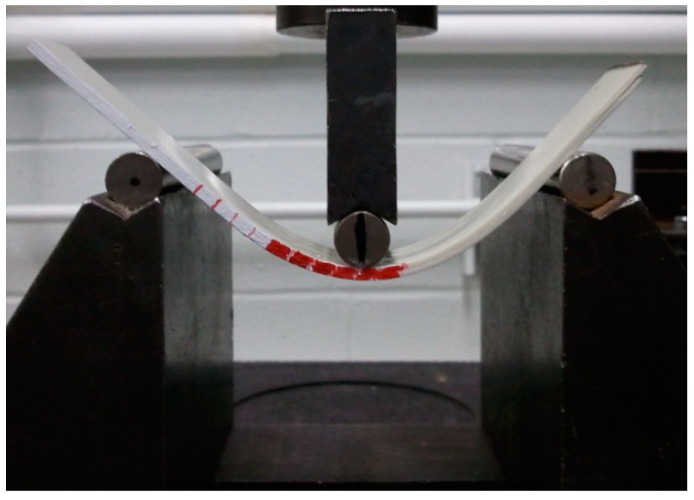
Experimental setup of end-notched flexure (ENF) testing.

Cured specimens with preinserted delamination were put into a three-point bending test fixture and made to undergo mid-span deflection at a rate of 2 mm/min on the grips. At the onset of delamination extension, the force on the loading cell was recorded while the crack was permitted to propagate (Figure 1).

During ENF testing, crack propagation occurs due to excessive shear deformation, thus exhibiting mode II failure with an abrupt nature. Such behavior makes it difficult to control the external load to achieve a target crack extension. Due to this limitation, only the first step of crack propagation during ENF testing was considered in the present study following ASTM D7905/D7905M [12].

**Figure 2:**
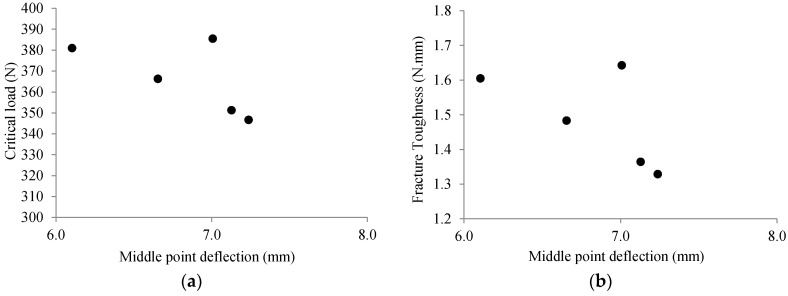
ENF results with a constant crack length (43 mm). (**a**) Variation of critical load versus middle point deflection and (**b**) variation of fracture toughness versus middle point deflection.

The results from ENF testing on five FRP samples are depicted in Figure 2. The points in Figure 2a correspond to the load and the middle point deflection at which delamination started to propagate for each sample. Mode II fracture toughness, $G_{IIC}$, (Figure 2b) was calculated based on the compliance method using the equation below [13]:

(1) $$G_{IIC}=\frac{9a^{2}P^{2}}{2w^{2}t^{3}E_{\mathrm{longitudinal}}}$$

where a is the initial crack length, P is the critical load at the onset of crack propagation, w is the sample width, t is the sample thickness, and $E_{\mathrm{longitudinal}}$ is the elastic modulus of the composite along the specimen length direction aligning with the fiber’s orientation. The elastic properties of these samples in different directions have already been measured and reported in [14], where the modulus of elasticity and elongation were found to be 44058.68 ± 3460.23 MPa and 7.93% ± 1.40% along the longitudinal direction and 1814.72 ± 697.73 MPa and 0.08% ± 0.03% along the transverse direction, respectively.

The variation in the test results demonstrates that the material fracture properties change from one sample to another due to uneven distribution of fibers, pressure, heating, and cooling during manufacturing as well as human error. Such behavior of UD laminated composites embraces the necessity of considering the randomness of properties in simulation studies of these materials.

## 3. XFEM Modeling of Delamination

In many cases during the early stages of product development, the structure’s dimensions and the test setup configurations make full-scale experimental evaluation cumbersome, increasing the demand for numerical analyses instead. A variety of numerical modeling techniques have been proposed in the past decades, and they can be categorized into mesh-free methods, such as smoothed particle hydrodynamics (SPH) [15], element-free Galerkin method (EFGM) [16], and finite difference method (FDM) [17], and mesh-based methods, such as finite element method (FEM) [18] and boundary element method (BEM) [19]. When it comes to modeling cracks, each method has its own advantages and disadvantages, and while the formulations of some techniques allow easier definition of cracks, e.g., SPH [20], others require modifications to their mathematical foundations so that analytical information about cracks can be included in the numerical space, e.g., FEM [21].

Various modifications to FEM have been proposed to account for crack nucleation and propagation. In cohesive zone models [22,23], it is assumed that a fracture process zone exists ahead of the crack tip, which develops according to a traction–separation law. With this approach, the stress singularity at the crack tip is avoided; however, its major limitation is that it requires prior knowledge of the crack paths for incorporating cohesive elements. Phase-field models [24,25] implicitly track cracks in the computational domain by introducing an auxiliary scalar field variable to represent crack topology. A governing equation defines the interaction between displacement and auxiliary parameter fields to model crack initiation and propagation. Another method is the coupled criterion framework [26,27], which combines energy-based and stress-based conditions for crack nucleation to improve the ability and efficiency of FEM in predicting crack initiation loading.

FEM can also be enhanced and used in modeling discontinuities by enriching its polynomial approximate functions, the so-called shape functions, with the partition of unity method (PUM) proposed by Melenk and Babuška [28]. This hybrid scheme is known as the extended finite element method (XFEM) [29,30], which offers a more accurate and efficient means to study the geometries and evolution of cracks in structures. In XFEM, similar to conventional FEM, the finite element mesh is generated regardless of the discontinuity locations. Then, specific search algorithms, such as the level-set or fast marching methods are utilized to identify the location of any discontinuity with respect to the existing mesh and apply enrichment on the affected elements. Next, additional auxiliary degrees of freedom are added to a group of nodes around the discontinuity. These degrees of freedom assist the model in capturing the displacement jumps caused by discontinuities.

Based on the XFEM modeling framework, we have previously developed a user-defined element in the finite element (FE) software Abaqus to simulate delamination in UD composites under mode I loading [31]. Here, in the next sections, we provide the fundamental formulations of our approach and demonstrate its efficacy to analyze mode II delamination failure.

### 3.1. Modeling Cracks in XFEM

**Figure 3:**
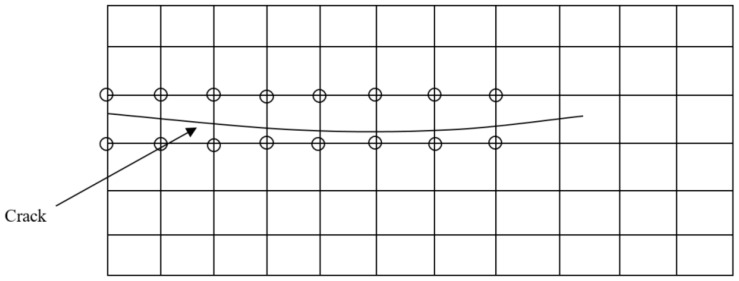
An arbitrary finite element mesh with a discontinuity (circles represent the enriched nodes of the mesh).

Assume a discontinuity (a crack) within an arbitrary finite element mesh (Figure 3). The displacement field of point X, u(X), inside the domain is described with two parts: one related to the conventional finite element approximation and the other associated with the XFEM enriched field defining the discontinuity [29]:

(2) $$u(X)=\underset{\begin{array}{c} i\\ n_{i}\in N^{all} \end{array}}{\sum }\phi_{i}\left(X\right)u_{i}^{\mathrm{ord}}+\underset{\begin{array}{c} j\\ n_{j}\in N^{f} \end{array}}{\sum }\phi_{j}\left(X\right)\psi \left(X\right)u_{j}^{\mathrm{enr}}$$

where $\phi_{i}\left(X\right)$ is the conventional shape function, ψ(X) is the general enrichment function, $N^{all}$ is the finite element mesh nodes, $N^{f}$ is the enriched nodes of the mesh, $u_{i}^{\mathrm{ord}}$ is the classic degrees of freedom at the node *i*, and $u_{j}^{\mathrm{enr}}$ is the additional enriched degree of freedom at the enriched node j.

**Figure 4:**
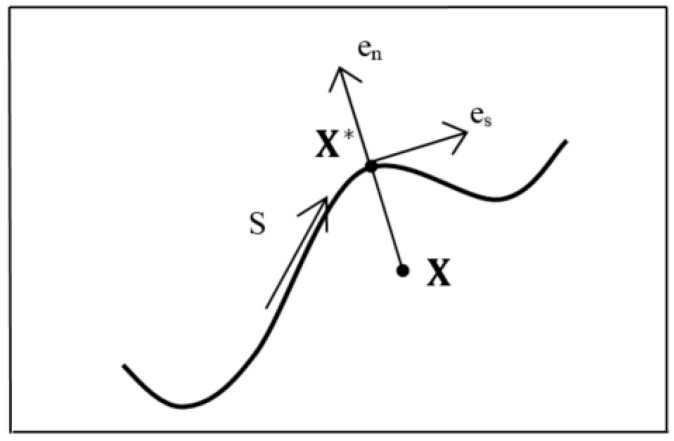
Unit tangential and normal vectors for the Heaviside function and nearest point to X on the crack surface; $X^{*}$.

In order to choose the enrichment function, i.e., ψ(X), any discontinuous function in the problem domain can be employed to estimate the displacement field approximation in the vicinity of the crack. A function that satisfies such a requirement is the Heaviside step function, H(X). It gains a value of +1 on one side of the crack and −1 on the other side and can be utilized when the crack propagation is modeled by multiple straight line segments. To find the Heaviside function value at each node of an element, tangential and normal vectors of the crack surface curve should be measured. If $X^{*}$ is the nearest point of a crack to the node X (Figure 4) and $e_{n}$ is the unit normal vector of the crack at point $X^{*}$ where $e_{s}\times e_{n}=e_{z}$ ($e_{s}$ is the unit tangential vector, and $e_{z}$ is the out of plane vector), then using a scalar product between the distance vector of the element’s nodes and the normal vector of the crack surface, the Heaviside function value can be calculated as follows:

(3) $$H\left(X\right)=\{\begin{array}{cc} +1, & if\;\left(X-X^{*}\right).e_{n}>0\\ -1, & otherwise \end{array}$$

### 3.2. Modeling Contact on Material Interfaces Using XFEM

During ENF testing, samples undergo large deformation and may experience plasticity (Figure 1). We therefore adopted a technique proposed by Khoei et al. [32] that integrates large deformation and contact modeling into XFEM formulations. Here, we provide the fundamental formulations for this technique and invite the reader to refer to [32] for detailed development of the model.

For nonlinear problems, implementation of a finite element method results in a set of nonlinear algebraic equations, R(u)=P, in which P is the external load and R is a nonlinear function of nodal displacements, u(X). This set of nonlinear equations is solved through an incremental iterative technique in which the problem is divided into incremental load steps, and a linear set of equations needs to be solved for each step, i.e., $K_{T}\Delta u=\Delta P$, where $K_{T}$ is the tangential stiffness matrix. In our nonlinear XFEM model, $K_{T}$ is defined as follows [32]:

(4) $$K_{T}=K_{Mat}+K_{Geo}=\int_{\Gamma_{V}}{\bar{B}}^{T}D_{S}^{ep}\bar{B}\;d\Gamma_{V}+\int_{\Gamma_{V}}G_{s}{}^{T}M_{S}^{}G_{s}\;d\Gamma_{V}$$

where $K_{Mat}$, $K_{Geo}$, $\bar{B}$, $D_{s}^{ep}$, $G_{s}$, and $M_{s}$ are matrices associated with materials stiffness, geometrical stiffness, strain gradient, contact constitute behavior, Cartesian shape function derivatives, and rearranged second Piola–Kirchhoff stress, respectively. $\Gamma_{V}$ also denotes the element domain. In addition to the conventional FEM part of nodal vectors, $\bar{B}$ and $G_{s}$ also includes enriched elements as given below:

(5) $$\bar{B}=\left[\begin{array}{cc} {\bar{B}}^{u^{\mathrm{ord}}} & {\bar{B}}^{u^{\mathrm{enr}}} \end{array}\right]$$

(6) $$G_{s}=\left[\begin{array}{cc} G_{s}^{u^{\mathrm{ord}}} & G_{s}^{u^{\mathrm{enr}}} \end{array}\right]$$

where ${\bar{B}}^{u^{\mathrm{enr}}}$ and $G_{s}^{u^{\mathrm{enr}}}$ are defined as follows (*H* is the Heaviside function) [32]:

(7) $${\bar{B}}^{u^{\mathrm{enr}}}=\left[\begin{array}{ccc} \frac{\partial \left(N_{i}H\right)}{\partial X}\frac{\partial x}{\partial X} & \frac{\partial \left(N_{i}H\right)}{\partial X}\frac{\partial y}{\partial X} & \frac{\partial \left(N_{i}H\right)}{\partial X}\frac{\partial z}{\partial X}\\ \frac{\partial \left(N_{i}H\right)}{\partial Y}\frac{\partial x}{\partial Y} & \frac{\partial \left(N_{i}H\right)}{\partial Y}\frac{\partial y}{\partial Y} & \frac{\partial \left(N_{i}H\right)}{\partial Y}\frac{\partial z}{\partial Y}\\ \frac{\partial \left(N_{i}H\right)}{\partial Z}\frac{\partial x}{\partial Z} & \frac{\partial \left(N_{i}H\right)}{\partial Z}\frac{\partial y}{\partial Z} & \frac{\partial \left(N_{i}H\right)}{\partial Z}\frac{\partial z}{\partial Z}\\ \frac{\partial \left(N_{i}H\right)}{\partial Y}\frac{\partial x}{\partial X}+\frac{\partial \left(N_{i}H\right)}{\partial X}\frac{\partial x}{\partial Y} & \frac{\partial \left(N_{i}H\right)}{\partial Y}\frac{\partial y}{\partial X}+\frac{\partial \left(N_{i}H\right)}{\partial X}\frac{\partial y}{\partial Y} & \frac{\partial \left(N_{i}H\right)}{\partial Y}\frac{\partial z}{\partial X}+\frac{\partial \left(N_{i}H\right)}{\partial X}\frac{\partial z}{\partial Y}\\ \frac{\partial \left(N_{i}H\right)}{\partial Z}\frac{\partial x}{\partial Y}+\frac{\partial \left(N_{i}H\right)}{\partial Y}\frac{\partial x}{\partial Z} & \frac{\partial \left(N_{i}H\right)}{\partial Z}\frac{\partial y}{\partial Y}+\frac{\partial \left(N_{i}H\right)}{\partial Y}\frac{\partial y}{\partial Z} & \frac{\partial \left(N_{i}H\right)}{\partial Z}\frac{\partial z}{\partial Y}+\frac{\partial \left(N_{i}H\right)}{\partial Y}\frac{\partial z}{\partial Z}\\ \frac{\partial \left(N_{i}H\right)}{\partial Z}\frac{\partial x}{\partial X}+\frac{\partial \left(N_{i}H\right)}{\partial X}\frac{\partial x}{\partial Z} & \frac{\partial \left(N_{i}H\right)}{\partial Z}\frac{\partial y}{\partial X}+\frac{\partial \left(N_{i}H\right)}{\partial X}\frac{\partial y}{\partial Z} & \frac{\partial \left(N_{i}H\right)}{\partial Z}\frac{\partial z}{\partial X}+\frac{\partial \left(N_{i}H\right)}{\partial X}\frac{\partial z}{\partial Z} \end{array}\right]$$

(8) $$G_{s}^{u^{\mathrm{enr}}}=\left[\begin{array}{ccc} \frac{\partial \left(N_{i}H\right)}{\partial X} & 0 & 0\\ 0 & \frac{\partial \left(N_{i}H\right)}{\partial X} & 0\\ 0 & 0 & \frac{\partial \left(N_{i}H\right)}{\partial X}\\ \frac{\partial \left(N_{i}H\right)}{\partial Y} & 0 & 0\\ 0 & \frac{\partial \left(N_{i}H\right)}{\partial Y} & 0\\ 0 & 0 & \frac{\partial \left(N_{i}H\right)}{\partial Y}\\ \frac{\partial \left(N_{i}H\right)}{\partial Z} & 0 & 0\\ 0 & \frac{\partial \left(N_{i}H\right)}{\partial Z} & 0\\ 0 & 0 & \frac{\partial \left(N_{i}H\right)}{\partial Z} \end{array}\right]$$

where (*X,Y,Z*) and (*x,y,z*) refer to material and spatial coordinates, respectively. The contact constitutive matrix in Equation (4) is defined as follows [32]:

(9) $$D_{S}^{ep}=\left[\begin{array}{c} {\bar{K}}_{11}\\ 0\\ 0 \end{array}\;\begin{array}{c} 0\\ {\bar{K}}_{22}\\ 0 \end{array}\;\begin{array}{c} 0\\ 0\\ {\bar{K}}_{33} \end{array}\right]$$

where ${\bar{K}}_{ii}$ is the penalty stiffness assigned to the local coordinates on the contact surfaces. ${\bar{K}}_{11}^{}$ provides the impenetrable characteristic to the normal direction of the crack plane, which follows the Kuhn–Tucker thresholds [33]:

(10) $$\delta_{n}\geq 0,\;\;P_{Contact}\leq 0,\;\;\left(\delta_{n}\right)\times \left(P_{Contact}\right)=0$$

where $\delta_{n}$ is the crack opening displacement, and $P_{Contact}$ contains the vector of contact forces.

The remaining terms in the constitutive matrix, ${\bar{K}}_{22}^{}$ and ${\bar{K}}_{33}^{}$, create the friction forces and prevent the contact surfaces from abrupt sliding. For these terms, standard static and dynamic friction laws can be applied to perform the analysis [32].

### 3.3. Cohesive Zone Implementation

**Figure 5:**
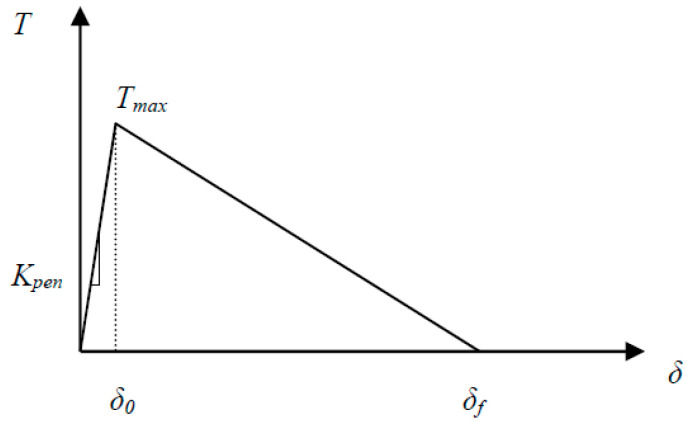
Bilinear traction–separation law for modeling material degradation.

As reported in previous studies [6,7], depending on the lay-up of the FRP composite, a large processing zone is expected to form during crack propagation. It is therefore necessary to incorporate cohesive crack modeling into XFEM formulations to analyze mode II delamination in FRP composites. Here, we have used a bilinear traction–separation law (Figure 5) that relates traction on crack faces, T, to mode I and mode II nodal displacements. In the bilinear form, the traction at the interface increases linearly with the crack tip opening displacement (CTOD) to a limit value, $T_{\max}$. The interface element then experiences softening until a traction of zero is reached, which corresponds to complete debonding. The total area enclosed by the bilinear curve represents the fracture toughness of the material.

To model the cohesive behavior ahead of the crack tip, a cohesive transformation matrix (${\bar{B}}_{Coh}^{}$) is used to relate crack normal opening and sliding displacements, δ, to the displacement of a point at the crack interface, $u^{k}$:

(11) $$\delta ={\bar{B}}_{Coh}\;u^{k}$$

In addition, the tractions on crack faces, T, is related to δ as defined below:

(12) $$T={\bar{D}}_{\mathrm{Interface}}\delta$$

where ${\bar{D}}_{\mathrm{Interface}}$ includes the cohesive interface material properties [6].

The cohesive transformation matrix can be extracted by finding the displacement in an enriched element. The displacement vector of a point in the enriched element, u(X), is as follow:

(13) $$u\left(X\right)=\underset{i}{\sum }\left(N_{i}u_{i}^{\mathrm{ord}}\right)+\underset{j}{\sum }\left(N_{j}\left(\underset{k}{\sum }N_{k}H_{k}-H_{j}\right)u_{j}^{\mathrm{enr}}\right)=\left[\begin{array}{cc} N & 0\\ 0 & N^{\mathrm{enr}} \end{array}\right]\left[\begin{array}{c} u^{\mathrm{ord}}\\ u^{\mathrm{enr}} \end{array}\right]$$

where

(14) $$N_{j}^{\mathrm{enr}}=N_{j}\left(\sum_{k}N_{k}H_{k}-H_{j}\right)$$

The conventional finite element shape function’s value remains constant for different points in the enriched element, while the enriched shape function’s value demonstrates an odd function property with respect to the interface position:

(15) $$N_{}^{\mathrm{enr}}\left(bottom\right)=-N_{}^{\mathrm{enr}}\left(top\right)$$

Thus, the global relative crack displacement, $\bar{\delta }$, can be described in the form of the displacement difference between two points above and beneath the crack surface:

(16) $$\bar{\delta }=u^{k}\left(top\right)-u^{k}\left(bottom\right)=\left[\begin{array}{cc} N & N^{\mathrm{enr}} \end{array}\right]\left[\begin{array}{c} u^{\mathrm{ord}}\\ u^{\mathrm{enr}} \end{array}\right]-\left[\begin{array}{cc} N & -N^{\mathrm{enr}} \end{array}\right]\left[\begin{array}{c} u^{\mathrm{ord}}\\ u^{\mathrm{enr}} \end{array}\right]$$

(17) $$\bar{\delta }=2\left[\begin{array}{cc} N & N^{\mathrm{enr}} \end{array}\right]\left[\begin{array}{c} u^{\mathrm{ord}}\\ u^{\mathrm{enr}} \end{array}\right]$$

In order to find the relative crack displacements in a global coordinate system, a simple transformation based on the normal and tangential directions, $m_{ij}$, of the crack plane with respect to the global coordinate can be employed:

(18) $$\delta =\left[\begin{array}{ccc} m_{11} & m_{12} & m_{13}\\ m_{21} & m_{22} & m_{23}\\ m_{31} & m_{32} & m_{33} \end{array}\right]\left[\begin{array}{c} {\bar{\delta }}_{X}\\ {\bar{\delta }}_{Y}\\ {\bar{\delta }}_{Z} \end{array}\right]=2\left[\begin{array}{ccc} m_{11} & m_{12} & m_{13}\\ m_{21} & m_{22} & m_{23}\\ m_{31} & m_{32} & m_{33} \end{array}\right]\left[\begin{array}{cc} 0 & N^{\mathrm{enr}} \end{array}\right]\left[\begin{array}{c} u^{\mathrm{ord}}\\ u^{\mathrm{enr}} \end{array}\right]={\bar{B}}_{Coh}\;u^{k}$$

Consequently, Equation (18) can be substituted into Equation (12) and used in the tangential stiffness formulation to introduce process zone properties within enriched elements:

(19) $$\begin{array}{l} K_{T}=K_{Mat}+K_{Geo}+K_{Coh}=\int_{\Gamma_{V}}{\bar{B}}^{T}D_{S}^{ep}\bar{B}\;d\Gamma_{V}+\int_{\Gamma_{V}}G^{T}M_{S}^{}G\;d\Gamma_{V}\\ +\int_{\Gamma_{c}}{\left({\bar{B}}_{Coh}\right)}^{T}{\bar{D}}_{Interface}{\bar{B}}_{Coh}\;d\Gamma_{c} \end{array}$$

Finally, in order to evaluate the internal forces, one can simply employ Equation (20) as follows:

(20) $$F_{\mathrm{int}}=\int_{\Gamma_{V}}{\bar{B}}^{T}\sigma \;d\Gamma_{V}+\int_{\Gamma_{C}}N^{\mathrm{enr}}{}^{T}f^{t}\;d\Gamma_{C}$$

Similar to conventional application of cohesive zone models, a predefined crack path was utilized to model the cracking behavior. With regard to the parameters in the traction–separation law, $T_{\max}$ was defined as 5.5 MPa based on the shear strength of PPS/glass composite samples [31] considering that the shear strength is 0.577 of tensile strength according to the maximum distortion energy theory. To identify an optimal value for $k_{\mathrm{pen}}$, a range of 10^3^ N/mm^3^ to 10^7^ N/mm^3^ was considered in trial simulations. It was observed that applying penalty stiffness lower than 10^4^ N/mm^3^ would result in extensive softening and a significant reduction in the peak load. Such behavior results from lower rigidity in the hardening region of the traction–separation law for the given material. On the other hand, excessive hardening was observed in simulations with $k_{\mathrm{pen}}$ higher than 10^6^ N/mm^3^, for which the deflection of the specimen reduced unrealistically and prevented the physical crack opening behavior from being modeled. A value of 10^5^ N/mm^3^ for penalty stiffness was eventually selected and used for subsequent simulations.

### 3.4. Stochastic Fracture Properties

Based on the experimental results given in Figure 2, we considered that the fracture properties of the tested UD samples had a stochastic nature as opposed to deterministic approaches where averaged values of experimental results are assumed for the estimation of material properties. The stochastic fracture properties of the materials were incorporated into our XFEM model through the procedure explained below.

**Figure 6:**
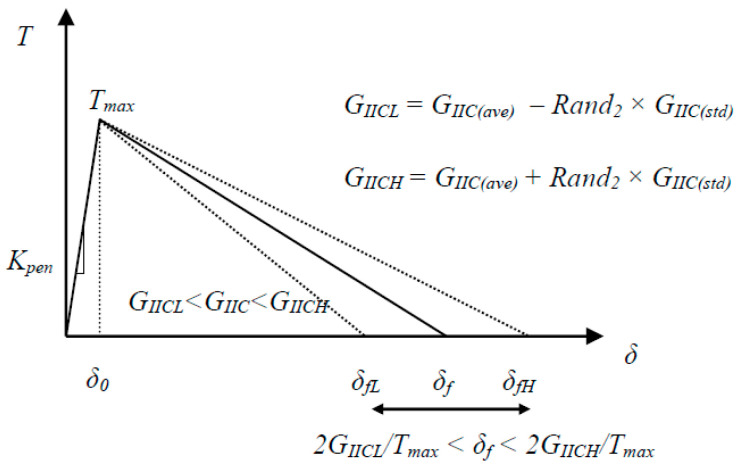
Proposed stochastic bilinear traction–separation behavior (*Rand*_2_ is a random number taken from a two-parameter Weibull distribution; *G_IICL_* and *G_IICH_* correspond to the lower and upper limits of *G_IIC_*. It was assumed that the onset of crack propagation was mainly influenced by the resin itself, and given that the resin showed more deterministic material properties, *T_max_* was assumed to have no variation in the model.

A random number within the range of experimentally measured mode II fracture toughness values ($G_{IIC}$) was picked to form a stochastic bilinear traction–separation law for the enriched elements in the cohesive zone (Figure 6):

(21) $$G_{IIC}=G_{IIC\left(ave\right)}+{\left(-1\right)}^{Rand_{1}}Rand_{2}\times G_{IIC\left(std\right)}$$

where $Rand_{1}$ is a random integer (odd or even to assign a random sign), and $G_{IIC\left(ave\right)}$ and $G_{IIC\left(std\right)}\;$ are average and standard deviations of fracture toughness, respectively. The second random number (*Rand*_2_), corresponding to individual enriched elements in each stage of damage evolution, was taken from a uniform distribution with a range of 0 to 1. The randomly selected values were then converted to a Weibull two-parameter distribution between 0 and 1 via the following formula:

(22) $$Rand_{2\;weibull}={\left[\frac{-1}{\alpha_{1}}\;\ln\left(1-Rand_{2\;uniform}\right)\right]}^{\frac{{}^{1}}{\beta_{1}}}$$

where $\alpha_{1}$ > 0 is a shape parameter, $\beta_{1}$ > 0 is the scale parameter of distribution, and both are considered to be equal to 3.

Because the fracture toughness distribution is considered to be a function of the crack length, a linear interpolation was utilized to extract *G_IIC_*_(*ave*)_ for each specific crack length. For *G_IIC_*_(*std*)_, it can be a constant or, in a more general form, it can be scaled with *G_IIC_*_(*ave*)_, which in turn becomes a function of crack length. It should also be added that according to the bilinear traction–separation law, a direct relationship exists between the critical fracture toughness, *G_IIC_*, critical sliding displacement, $\delta_{f}$, and maximum interface shear strength, $T_{\max}$:

(23) $$G_{IIC}=\frac{T_{\max}\delta_{f}}{2}$$

Therefore, the obtained statistical distribution of the fracture toughness can be converted into the variation of failure crack sliding and/or maximum interface strength of material via Equation (23). Khokhar et al. [34] introduced randomness into their simulation by implementing a relationship between random fracture toughness and the maximum interface strength by keeping the failure crack sliding displacement constant. To improve the convergence of the numerical simulation, the present study assumed a constant value for the maximum interface shear strength ($T_{\max}$= 5.5 MPa), while the failure crack sliding displacement was randomly varied during damage evolution (see Figure 6). This approach relies on constant penalty stiffness and prevents the over-strengthening of the stiffness of elements in the process zone. It also stands with the fact that the crack length extension in test specimens is a function of the CTOD and the energy release rate in front of the crack tip. Here, the process zone size was selected based on experimental results. Sensitivity analysis was performed with a range of process zones from 15 to 30 mm (length of approximately 15 to 30% of specimens) to confirm the assumption. A value of 25 mm for the cohesive zone length was used for stochastic simulations [35].

## 4. Results and Discussion

The above formulations were integrated into an Abaqus user-defined element subroutine, which is accessible in [14], coupled with MATLAB. The implicit solver of Abaqus was used for accurate evaluation of displacement field. Randomness was introduced into the analysis by means of the constant standard deviation method. In each simulation, a preassigned initial crack was considered in the specimen, and the stochastic fracture properties were assigned to the elements in the area near the crack front. Once fracture toughness value was assigned to a given element, it remained unchanged during the given simulation run. As the crack propagated, new elements would enter into the process zone and stochastic fracture properties would similarly be assigned to them.

**Figure 7:**
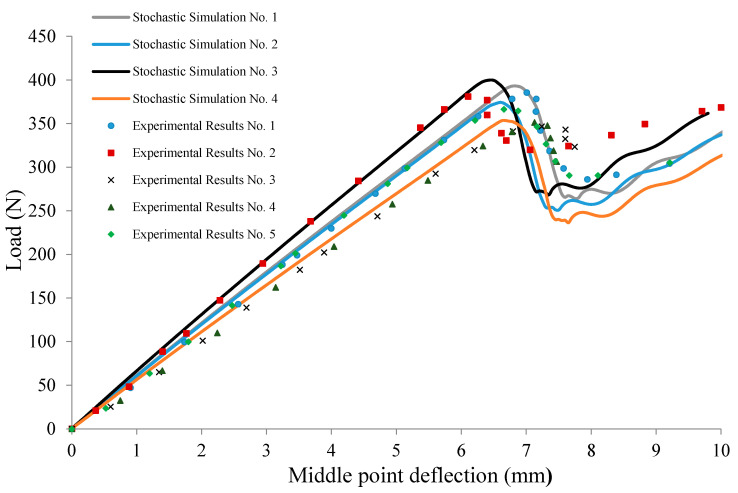
Comparison of stochastic measured and predicted force–displacement values in ENF tests on the polyphenylene sulfide (PPS)/glass samples.

**Figure 8:**
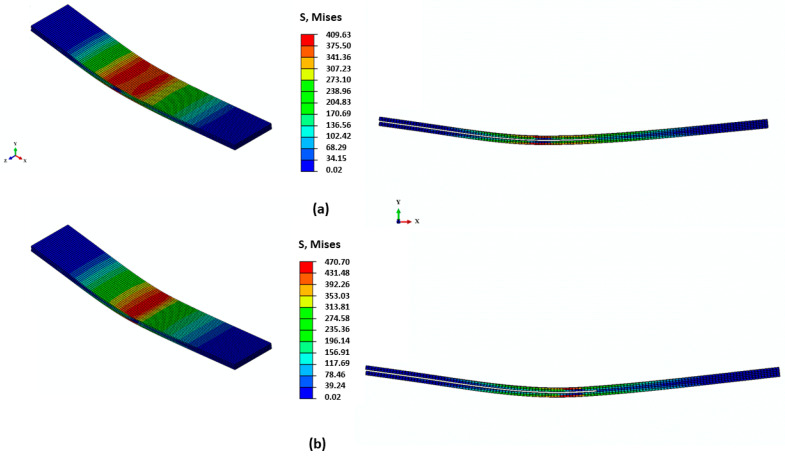
Stages of delamination propagation within the ENF numerical model. (**a**) Stress concentration at onset of crack propagation. (**b**) Stress distribution during degradation in traction–separation law. Note that interface is considered here as an equivalent (macro) medium, dominated by the resin along with random fiber bridges; notice the stress concentration at the crack front.

The results for continuous delamination are given in Figure 7. The results of the stochastic simulations were in great agreement with the nonrepeatable experimental data obtained from ENF testing. The critical sliding displacement, $\delta_{f}$, was different in each simulation run, affecting the value of the maximum load at which the mid-plane crack started to propagate. However, the shape of the load–displacement curve was the same irrespective of the $\delta_{f}$ value. Based on the trend in the simulation data, it can be concluded that the fiber bridging effect in ENF testing had minimal effect on the global characteristics of the response. Figure 8 shows the XFEM model contours under different stages of delamination during ENF testing. It should be noted that, from an application perspective, particularity in the context of composite-forming processes, the mode II loading, similar to ENF testing, would be more relevant due to the likelihood of sliding between layers of the laminate under the punch load.

## 5. Conclusion

In the present work, a framework is presented to numerically simulate the mode II fracture behavior of FRP composite materials. For this purpose, extending the capability of modeling delamination and contact interfaces in large deformation problems, a user-element subroutine was developed in Abaqus by incorporating nonlinear XFEM element properties with the cohesive zone model and contact formulation. In addition, the stochastic fracture properties of the composite samples were incorporated into the code to capture the randomness in properties evidenced in nonrepeatable ENF testing results of composite materials. The efficacy of the model was demonstrated by predicting the stochastic fracture behavior of PPS/glass UD laminates, which was in good agreement with the experimental data.
